# Impact of Technology Innovation on Air Quality—An Empirical Study on New Energy Vehicles in China

**DOI:** 10.3390/ijerph18084025

**Published:** 2021-04-12

**Authors:** Haoxuan Hu, Yuchen Zhang, Xi Rao, Yinghua Jin

**Affiliations:** 1 College of Materials Science and Engineering, Chongqing University, Chongqing 400045, China; hhx17815155067@163.com; 2 College of Architecture and Urban Planning, Chongqing University, Chongqing 400045, China; laurie_zyc@163.com; 3 College of Economics and Business Administration, Chongqing University, Chongqing 400045, China; 4 Sino-US College, Beijing Institute of Technology, Zhuhai 519088, China; ecoyjx@yahoo.com

**Keywords:** new energy vehicles (NEVs), technology innovation, air pollution, vehicle and vessel tax (VVT)

## Abstract

China’s high economic growth has been accompanied by deteriorating air quality in recent decades. This paper aims to explore the relation between technology innovation (defined as the invention patent counts of each region) of new energy vehicles (NEVs) in China and air quality. A panel fixed effect model is used to analyze this relation and the mediating effect methods are used to examine the role of the output of NEVs (defined as the annual production quantity of NEVs in each region (unit: thousand)). The results of our study show: (1) the impact of the technology innovation of NEVs on air quality is positive and statistically significant; (2) the mediating role of the output of NEVs is confirmed in the relation between NEVs innovation and air quality improvement; (3) the technology innovation of NEVs has a more notable impact on the air quality in the regions with higher vehicle and vessel tax (VVT). The present study implicates for the first time that the technology innovation of NEVs can enhance air quality with the mediating role of the output of NEVs and the moderating role of VVT.

## 1. Introduction

In recent years, with the rapid development of the economy, the air pollution has increased simultaneously in China [1,2]. The continuous improvement of air quality is an urgent requirement to achieve the ecological civilization development goals of carbon peak and carbon neutrality. Air pollution not only leads to multiple adverse health outcomes [3,4,5], but also has a negative effect in economic growth [6]. Improving air quality has become a significant task and a persistent issue [1,3,7]. As a result, more and more strategies and tactics have been formulated and implemented in response to air pollution [2]. Therefore, the improvement of air quality not only needs the support of government policies and the public’s awareness of environmental protection, but also depends on the technology innovation of manufacturing to reduce pollutant emissions [7], because exhaust emission from manufacturing is an important factor that contributes to air pollution [7]. China is one of the world’s largest emitters of pollutant emissions [4]. To reduce pollutant emissions and improve air quality, China has implemented the policy portfolio about the technology innovation of manufacturing, including the automotive industry as an example in regard to the reduction in exhaust emissions and utilization of clean energy [8].

Scientists have been dedicated to exploring innovation and new technology in automobiles and substituting new energy vehicles (NEVs) for traditional energy vehicles to improve air quality [9]. Past studies have shown some evidence that the innovation of hybrid and plug-in NEVs can effectively alleviate CO_2_ emissions [9,10,11], and renewable energy technological innovation can reduce air nitrogen dioxide concentrations [12].

Furthermore, previous studies have shown that the technology innovation for NEVs have triggered a massive growth in the production and purchase of NEVs, which can improve air quality by replacing traditional energy vehicles [13,14,15].

However, this mediating role of the output of NEVs in improving air quality through technology innovation in NEVs has not been quantified. Our study is to fulfil this task.

Some studies show that vehicle and vessel tax (VVT) contributes to technological innovation [12] and optimizes air quality [12]. Some provincial governments in China have implemented a higher VVT to reduce the emissions of traditional energy consumption vehicles and support the NEV industry [16,17]. NEV companies in these regions may be incentivized to focus on the technology innovation of NEVs thereby expanding sales, which is conducive to improving the air quality [18,19]. Therefore, the impact that the technology innovation of NEVs has on air quality is more notable in regions with a higher VVT than those with a lower VVT.

To test the stationarity of the panel data next, based on the mediating effect models, our study analyzed that the technology innovation of NEVs (defined as the invention patent counts of each region) could enhance air quality through total output of NEVs (defined as the annual production quantity of NEVs in each region, unit: thousand). Then, we found that the impact of technological innovation of NEVs on air quality is stronger in high tax regions compared with low tax regions. This indicates that increasing VVT significantly enhances the positive effect of technology innovation in NEVs on air quality. In the analysis of the above relations, the number of invention patents for the NEV industry is used to quantify technology innovation, and the annual number of days of excellent air quality in each provincial administrative region is used to quantify this region’s air quality.

The previous studies only focus on some specific kinds of NEVs or treat all kinds of patents evenly in measuring new energy technology innovation [9,10,16]. Up until now, to our best knowledge there have been no studies using all NEVs as a whole industry together with the output of NEVs and VVT to explore the relationship between technology innovation and air quality. In addition, it is generally accepted that invention patents, among patents for invention, patents for utility model and patents for design, have the strictest assessments requiring both prominent substantive features and notable progress [20]. Therefore, we only single out invention patent data as a measurement of technology innovation rather than all patent data used by the previous literature. Our study contributes to the literature and public policy with empirical evidence in three aspects: (1) analyzes the direct relationship between innovation of NEVs as a whole industry and air quality for the first time; (2) explores the mechanisms of relationship between NEVs’ technology innovation and air quality, with the mediating role of output of NEVs and the moderating role of VVT; (3) affirms that governments should lay emphasis on levying tax appropriately to keep a balance between technology innovation of NEVs and air quality improvement.

This paper is organized as follows: Section 2 discusses the role of technological innovation of NEVs in improving air quality, and develops three hypotheses to test; Section 3 describes the data and model; Section 4 reports and discusses empirical results; and Section 5 concludes.

## 2. Literature Review and Hypothesis Development

### 2.1. Technology Innovation of NEVs and Air Quality

With the number of vehicles increasing rapidly [21], vehicle-related pollutants have become the main source of air pollution in a lot of cities [22,23,24], contributing to 10%–15% of the total emissions in CO_2_ [25]. Thus, the improvement of air quality has continuously been a hot topic in recent years. At the same time, new energy technology innovation in vehicles is an important factor for reducing air pollution, which have been advocated by several international organizations such as the United Nations and WHO. [26,27].

NEVs are an important technological innovation by themselves, which can reduce the manufacturing and use of traditional fossil fuel vehicles to improve air quality [28,29]. A few studies have taken the output of NEVs into consideration, which indicates that there is some relationship between the technology innovation of NEVs and the output of NEVs [30,31]. For example, Wu proves that leading technology firms are more likely to attract more consumers, and the increase in vehicle purchases will stimulate the production of NEVs [27]. This idea has also been echoed by some international community proposals: the Paris Climate Accord indicated that the output of global NEVs should significantly increase to fight against the air pollution that human beings are facing [25]; the International Energy Agency (IEA) called for 140 million NEVs globally by 2030 to improve air quality [26].

Therefore, we tend to investigate the relation between technology innovation of NEVs and air quality, with the mediating role of NEVs output as well. When quantifying regional technological innovation, according to the previous literature, the number of patents is conventionally chosen as a typical indicator [20,32,33]. For example, Johnstone investigated the correlation between renewable energy policies and technological innovation using patent counts [32]. Zhu explored the impact of renewable energy technology innovations (RETIs) on climate change with patent counts as the measure of RETI [33]. Lin and Xiao found the functioning mechanism and effects of air pollution on technological innovation whose proxy was the patent counts [20]. Compared to utility model and design patents, invention patents are commonly the most technological complex and time-consuming type of intellectual property rights (IPRs). Invention patent filings and registrations typically require a more stringent level of innovation [20]. It is one of our paper’s improvements that we single out invention patent data as a measurement of technology innovation rather than all patent data used by the previous literature. Therefore, we believe that the invention patents of NEVs are more effective in reducing emissions and improving environmental friendliness.

Further, according to the results of search into invention patents, we find that more than 70 percent of NEVs’ invention patents are related to the technology of air quality improvement. In this study, we use the number of invention patents of the NEV industry to measure the technology innovation of NEVs.

Accordingly, we propose two hypotheses:

**Hypothesis** **1** **(H1).** *Technology innovation of the NEV industry is positively correlated with air quality.*

**Hypothesis** **2** **(H2).** *Technology innovation of the NEV industry correlates with air quality improvement through the mediating role of NEVs’ output.*

### 2.2. VVT and Air Quality

It has been evidenced that implementing environmental regulations is an effective solution to improve air quality [18]. Chinese governments at all levels have been formulating a series of environmental policies to promote the utilization of NEVs [22,23], such as tax exemption on NEVs [11,13].

NEVs have been exempted from VVT [16,18,19], and this tax exemption policy has a positive effect on air quality [23]. According to the modified VVT policy in 2012, the annual benchmark tax on passenger vehicles and vessels ranges from 60 yuan for less than 1.0 L discharge volume to 5400 yuan for more than 4.0 L discharge volume [16]. Such a wide gap encourages more consumers to buy NEVs or those traditional vehicles with low energy consumption and low emissions [18]. It is notable that different regions have different standards of VVT according to the local conditions (Appendix A). Those regions with a higher VVT indicate that governments have more firm determination to control the emissions of traditional energy consumption vehicles and improve the air quality [17,19]. Thus, NEV companies in these regions may be pushed to act more on the technology innovation of NEVs [16,18].

As elaborated above, we suggest that VVT plays a moderating role in the connectivity from the technology innovation of NEVs to air quality improvement. Furthermore, this research attempts to explore whether different standards of VVT have different effects on NEVs’ impact on air quality.

Therefore, the following hypothesis is proposed.

**Hypothesis** **3** **(H3).** *VVT can enhance the positive relationship between the technology innovation of NEVs and air quality.*

## 3. Research Design

### 3.1. Data Acquirement and Variables

In this paper, our study period is from 2014 to 2019, and all of the data are provincial level based on the data availability. The number of invention patents of the NEV industry in 31 provincial administrative regions of mainland China is used as a proxy for renewable energy technology innovation (RETI) to quantify technology innovation of NEVs. This data is retrieved from Wanfang Database (http://www.wanfangdata.com.cn/index.html, accessed on 20 January 2021). Additionally, during the period of our study (2014–2019), the number of excellent air quality days (AQI < 100) annually is used to quantify air quality in each provincial administrative region from the source: China Stock Market and Accounting Research Database (CSMAR).

According to the IPAT theory [34], other explanatory variables such as the population, the proportion of the secondary industry in GDP, the forest coverage rate and the sum of VVT potentially have an impact on air quality of a region. Therefore, we take them as the control variables. The data for them are from CSMAR database.

### 3.2. Empirical Model

We used *STATA 16.0* to run the regressions based on the following model:

(1) $$AQI_{i,t}=\alpha \ln Patent_{i,t}+\underset{i,t}{\sum }\beta_{i,t}Control_{i,t}+\varepsilon_{i,t}$$

In Model 1, α and β are the regression coefficients, *AQI_i,t_* represents the air quality of the *i* region in *t* year, *Patent_i,t_* represents the technology innovation of NEVs of the *i* region in *t* year, *Control_i,t_* represents the collection of control variables of the *i* region in *t* year, and *ε_i,t_* is the error term of the regression model of the *i* region in *t* year.

## 4. Empirical Results

This study attempts to quantify the impact that the technology innovation of NEVs has on air quality in China by the mediating role of the output of NEVs and the moderating role of VVT.

### 4.1. Descriptive Statistics

Table 1 is a description of statistics about the data. We summarize the panel data of 31 provincial administrative regions in mainland China during 2014–2019, such as the number of days that have excellent air quality status, the number of NEV invention patents per year, the population, the proportion of GDP in the secondary industry, the sum of VVT, the forest coverage rate, the output of NEVs and the VVT charging criteria in each provincial administrative region.

As shown by Table 1, the indicators such as mean values, standard deviations (SD), minimums and maximums of each variable are listed. The variations of the data for most variables are very large. For example, the SD values of AQI and Patent are 60.700 and 70.660, respectively. Given that large deviation may bias our results, we used the natural logarithm of the value of invention patents of NEVs in our estimation. Table 2 presents pairwise correlations between all variables in this research. All correlation coefficients are lower than 0.4. Pearson’s correlations of all variables are on the bottom left corner of the table and Spearman’s correlations on the top right corner.

To test the stationarity of the panel data, we performed the Breitung panel unit root tests according to Breitung (2000) [35]. The null hypothesis is that a variable contains a unit root (i.e., it is not stationary). The estimated results for major interesting variable data are reported in Table 3.

Table 3 includes level results of the unit root tests and all data series are stationary with high statistical significance in I (0). Therefore, we can soundly reject the null hypothesis of the panel unit root. This stationarity evidence probably also echoes the statement when the panel individual number N (N = 31 in this study) is larger than the time series number T (T = 6 in this study), the stationary testing is not required and usual panel data procedures are recommended [36].

After the Breitung panel unit root tests, we performed the Granger causality tests. The results are reported in Table 4. In the first line, we found that Patent can be regarded as the Granger cause of AQI. In the second line, the union significance of the coefficient of the test variable (AQI) is with the statistic chi-square of 0.066 and the relevant *p*-value is 0.798. It indicates that AQI cannot be regarded as the Granger cause of Patent. To sum up, the evidence suggests that the technology innovation of NEVs represented by invention patent counts Granger causes the air quality improvement measured by AQI, but not vice versa.

### 4.2. Baseline Results

Before the baseline analysis, we carried out a further search to the invention patents in the NEVs industry, finding that more than 70% of all invention patents are directly related to the technology of air quality improvement. For example, one patent concerns the pollution prevention in mold making of new energy vehicles manufacturing. The remaining 30% of (at most) invention patents are not so relevant to air quality, which focus on the mechanistic designing of production efficiency improvement, the reduction in production cost of vehicle parts, etc.

Table 5 reports the panel fixed effect regression results for the relationship between the technology innovation effect of NEVs and AQI. The dependent variable is AQI, being measured as days of excellent air status. The independent variable is the technology innovation of NEVs, being measured as the number of invention patents of NEVs. The variable definitions are shown in Table 1. Robust *t*-statistics are reported in parentheses. The estimates of the two models are highly statistically significant, indicating that the value of invention patent counts is one of contributors for emissions reduction and environment protection. According to the Hausman test (chi-square = 10.440, *p* = 0.001), we chose to use fixed effects regression models for data analysis. In column (1), we only include the interesting variable with yearly fixed effects and provincial fixed effects. The estimated coefficient of the interesting variable: Patent, is 1.102 and statistically significant at the 0.1% level, suggesting that the technology innovation of NEVs is positively correlated with excellent air quality days. In column (2), we add controls in our estimation. The coefficient on Patent is still positive and statistically significant at the 0.1% level. It suggests that the causal relationship between Patent and AQI is robust. Our results are basically similar to those of the previous studies [37]. Overall, our results are consistent with Hypothesis 1 and support the positive association between the technology innovation of NEVs and AQI [37,38], indicating that the technology innovation of NEVs can enhance air quality in China.

### 4.3. Analysis of Mediator Effect

Mediator analysis is a set of statistical methods used to investigate whether a variable has an intermediary structure [37]. According to the previous study [39], mediation is tested with the causal step procedures (Baron and Kenny, 1986), in which the relationships among the independent variable, dependent variable and the mediator variable are analyzed as follows: (a) the dependent variable is regressed on the independent variable; (b) the mediator variable is regressed on the independent variable; (c) the dependent variable is regressed on both the independent variable and mediator variable. These regression equations can be written in Model 1, 2 and 3. We suggest that the output of NEVs is the mediator variable.

In Model 1, 2 and 3, we tested the mediating effect of the output of NEVs on the process where the technology innovation of NEVs influences air quality. The direct effect of technology innovation on air quality remains statistically significant after including the mediator variable in Model 1 (α = 1.034, *p* < 0.001), which has been reported in the baseline results of Table 5. In Model 2, Output is statistically significant and positively associated with Patent (α′ = 0.673, *p* < 0.001), which indicates that the technology innovation of NEVs enhances the output of NEVs. In Model 3, there is a significant association in AQI with Patent and Output (α″ = 0.019 < α, *p* < 0.001), which indicates the statistically significant mediating effect by the output of NEVs between technology innovation and air quality. The estimates of panel fixed effect regression are shown in Table 6.

(2) $$Output_{i,t}=\alpha^{\prime }\ln Patent_{i,t}+\sum_{i,t}\beta_{i,t}^{\prime }Control_{i,t}+\mu_{i,t}^{\prime }$$

(3) $$AQI_{i,t}=\alpha^{\prime \prime }\ln Patent_{i,t}+\sum_{i,t}\gamma_{i,t}Output_{i,t}+\sum_{i,t}\beta_{i,t}^{\prime \prime }Control_{i,t}+\mu_{i,t}^{\prime \prime }$$

### 4.4. Analysis of Moderator Effect

Appendix A of this paper lists the indicators of vehicle and vessel tax, abbreviated as VVT in 31 provincial administrative regions (yuan/year) and the preferential policies about VVT in mainland China.

According to the VVT in each provincial administrative region, we select the tax rate standard of 1.0–1.6 L (the mainstream vehicle type that accounts for about 70 percent of total automobile sales in China [39]) as the moderator variable. As shown in Appendix A, this tax varies in each provincial administrative region, with an average of 335.5 yuan, a maximum of 420 yuan and minimum of 300 yuan.

Therefore, in order to better understand the role that VVT plays, the whole sample was divided into two groups (low tax group and high tax group) based on the tax rates of VVT. Between them, the low tax group is that the tax rates are less than the median (350 yuan/L), consisting of 17 provincial administrative regions. The other group is that the tax rates are more than 350 yuan/L, which includes 14 provincial administrative regions. We ran regressions of Model 1 for both groups. In the low tax group, only three control variable estimates were statistically significant while in the high tax group five variable estimates were statistically significant (including the independent variable).

The results of panel fixed effect regression analysis of both groups are shown in Table 7.

In Table 7, the estimates of VVT are not statistically significant in low tax regions (only three control variables significant with *p* < 0.01). However, they become statistically significant in high tax regions with independent variables and five control variables are also statistically significant (*p* < 0.001). In addition, we ran a seemingly unrelated regression estimation to examine the difference of significances between the above two groups, with results of difference = 0.908 and chi-square = 5.12, meaning the two groups are statistically significantly different. Therefore, we can conclude that VVT is a moderator variable between the technology innovation of NEVs and air quality. VVT can moderate the association between the technology innovation of NEVs and air quality in China.

### 4.5. Robustness Test

Lastly, we perform a robustness check by singling out the air-quality-related invention patents and regressing *AQI* on the air-quality-related invention patents only. The results are reported in Table 8. The difference between the results in Table 5 and the results in Table 8 is that *Patent* in Table 5 includes all invention patents of NEVs while Patent in Table 8 includes only the invention patents directly related to air quality. Based on the results, we can see clearly that when including invention patents directly related to air quality, the significance of our variable estimates in regressions even rises, with higher absolute values of coefficients. This indicates that the invention patents closely correlate with emissions reduction and being environment friendliness.

## 5. Conclusions

Nowadays, the control of environmental pollution has become a hot and prioritized issue in China and abroad. In this paper, we used a panel fixed effect model to analyze the data from 31 provincial administrative regions in mainland China to test our three hypotheses: (1) the technology innovation of NEVs industry is positively correlated with air quality; (2) the technology innovation of NEVs industry correlates with air quality improvement with an impact path of NEVs’ output; and (3) VVT can enhance the positive relationship between the technology innovation of NEVs and air quality.

This study finds that these influencing factors improve the air quality statistically and economically significantly. The relevant policy implications that can be derived from this study are as follows.

For the automotive manufacturing industry, the companies in this industry should increase expenditures on research and development in pursuit of state-of-the-art technological innovation which addresses air quality and environmental friendliness. A massive replacement of traditional energy vehicles with NEVs having frontier technologies can be popularized to reduce pollution emissions. For relevant government agencies and departments at all levels, this study provides strong empirical evidence in China to appropriately increase VVT rates, because VVT can incentivize consumers to substitute traditional energy vehicles with NEVs. At the same time, central and local governments can also enact a series of preferential tax treatments to the vehicle manufacturers that have invested heavily in NEV research and development.

## Figures and Tables

**Table 1:** Descriptive statistics.

Variables		Definition	Mean	SD	Min	Median	Max
Dependent Variable	AQI	Number of days of air quality excellent status (day)	263.200	60.700	93	260	362
Independent Variable	Patent	Number of NEVs invention patent (piece)	26.330	70.660	0	3	523
Control Variable	POP	Population (ten thousand)	4954	7540	317.600	3811.700	100,047
	2nd Industry	The proportion of GDP in the secondary industry (%)	41.350	7.844	16.200	42.584	54.140
	VVT Sum	Sum of VVT (ten thousand yuan)	232,409	176,065	7647	181,762	843,520
	Forest	Forest coverage rate (%)	33.000	18.090	4.240	35.840	66.800
Mediator Variable	Output	Output of NEVs (ten thousand)	2.389	3.155	0	1.100	15.440
Moderator Variable	VVT	1–1.6 L emissions VVT in 31 provinces (yuan)	335.500	43.960	300	350	420

NEV: new energy vehicle; VVT: vehicle and vessel tax; AQI: air quality; POP: population.

**Table 2:** Pearson’s correlations and Spearman’s correlations of variables.

	AQI	Patent	POP	2nd Industry	Sum	Forest	Output	VVT
AQI		0.317 ***	0.067 **	0.079 **	0.070 ***	0.147 **	0.191 **	0.076 ***
Patent	0.377 ***		0.017 ***	−0.199 **	−0.313 **	−0.297 ***	0.208 *	−0.203 ***
POP	0.045 *	0.022 **		0.217 **	−0.305 *	0.312 **	0.171 ***	0.509 **
2nd Industry	0.063 **	−0.225 *	0.123		0.204 **	0.355 ***	0.041 **	0.336 **
VVT Sum	0.057 *	−0.253 **	−0.170 **	0.140 **		0.102 *	−0.025 *	0.082 *
Forest	0.133 ***	−0.376 *	0.126 ***	0.394 *	0.078 *		0.216 ***	0.578 **
Output	0.212 **	0.394 *	0.166 ***	0.028 **	−0.038	0.253 ***		0.149 *
VVT	0.088 **	−0.144 ***	0.388 *	0.357 *	0.095 **	0.320 *	0.123	

Note: Robust *t*-statistics are reported in parentheses. *, **, and *** indicate statistical significance at the 10%, 5%, and 1% levels, respectively.

**Table 3:** Breitung panel unit root tests.

Variables	Constant
AQI	−0.909 ***
	0
Patent	−1.023 **
	−0.005
Output	−1.003 **
	−0.002
VVT	−2.077 **
	−0.006
2nd Industry	1.579 *
	−0.002
POP	1.958 ***
	0
Forest	2.905 ***
	0

Note: ***, ** and * indicate stationarity at 1%, 5%, and 10% significance levels, respectively. *p*-value are reported in parentheses.

**Table 4:** Granger causality Wald tests.

Equation	Excluded	Chi-Square	Prob > Chi-Square
AQI	Patent	7.812	0.005
Patent	AQI	0.066	0.798

**Table 5:** Innovation and AQI—baseline analysis.

Dependent Variable	AQI	(1)	(2)
Independent Variable	Patent	1.012 ***	1.034 ***
		(2.741)	(2.973)
Control Variable	POP		0.010 *
			(1.900)
	Forest		−0.017 **
			(−2.305)
	2nd Industry		−0.011 **
			(−2.597)
	VVT Sum		−0.103 *
			(−1.896)
Constant		−71.884 ***	−59.552 *
		(−5.366)	(−1.694)
Year FE		YES	YES
Province FE		YES	YES
Observations		186	186
Adj.R^2^		0.360	0.336

Note: Robust *t*-statistics are reported in parentheses. *, **, and *** indicate statistical significance at the 10%, 5%, and 1% levels, respectively; FE: fixed effect.

**Table 6:** Panel fixed effect regression results of Model 2 and Model 3.

Dependent Variable	Model 2		Model 3	
	Output		AQI	
Independent Variable	Patent	0.673 *** (5.565)	Patent	0.019 *** (2.923)
			Output	0.023 *** (3.015)
Control Variable	POP	0.017 ***	POP	0.031 **
		(2.918)		(2.336)
	Forest	−0.002	Forest	0.007 ***
		(−0.195)		(8.824)
	2nd Industry	0.043 *	2nd Industry	−0.006 ***
		(1.819)		(−2.993)
	VVT Sum	0.983 ***	VVT Sum	−0.157 ***
		(4.045)		(−8.347)
	Constant	−12.636 ***	Constant	7.384 ***
		(−4.487)		(33.459)
Year FE		YES	Year FE	YES
Province FE		YES	Province FE	YES
Observations		186	Observations	186
Adj.R^2^		0.319	Adj.R^2^	0.385

Note: Robust *t*-statistics are reported in parentheses. *, **, and *** indicate statistical significance at the 10%, 5%, and 1% levels, respectively.

**Table 7:** VVT moderator effect test results in two groups.

Dependent Variable		AQI	
		Low Tax Group	High Tax Group
Independent Variable	Patent	0.017	8.622 ***
		(1.633)	(3.027)
Control Variable	POP	0.001 *	−15.451
		(1.882)	(−1.265)
	Forest	0.003	1.877 ***
		(1.913) *	(7.619)
	2nd Industry	−0.017 ***	−57.865 ***
		(−3.235)	(−3.297)
	VVT Sum	−0.362	−29.897 ***
		(−1.556)	(−2.758)
	Constant	−75.244	783.878 ***
		(−1.264)	(5.070)
Year FE		YES	YES
Province FE		YES	YES
Observations		102	84
Adj.R^2^		0.394	0.441
Difference		0.908	
Chi-square		5.12 ***	

Note: Robust *t*-statistics are reported in parentheses. *, and *** indicate statistical significance at the 10%, and 1% levels, respectively.

**Table 8:** Innovation and AQI—robustness test (only air quality directly related invention patents included).

Dependent Variable	AQI	(1)	(2)
Independent Variable	Patent	1.725 ***	1.116 ***
		(2.945)	(2.790)
Control Variable	POP		0.009 *
			(1.913)
	Forest		−0.020 **
			(−2.311)
	2nd Industry		−0.016 **
			(−2.479)
	VVT Sum		−0.121 *
			(−1.877)
Constant		−60.894 ***	−52.769 *
		(−4.277)	(−1.701)
Year FE		YES	YES
Province FE		YES	YES
Observations		186	186
Adj.R^2^		0.323	0.307

Note: Robust *t*-statistics are reported in parentheses. *, **, and *** indicate statistical significance at the 10%, 5%, and 1% levels, respectively.

## Data Availability

The data used in this study were acquired from https://www.wanfangdata.com.cn/index.html (accessed on 20 January 2021) and https://www.gtarsc.com/ (accessed on 20 January 2021).

## Appendix A

**Table A1:** The indicators of VVT in 31 provincial administra-tive regions (yuan/year) and the preferential policies about VVT in mainland China.

Type	Province	<1 L	1–1.6 L	1.6–2 L	2–2.5 L	2.5–3 L	3–4 L	>4 L
Low Tax Regions	Chongqing	120	300	360	660	1200	2400	3600
	Ningxia	120	300	360	660	1800	3000	4500
	Qinghai	60	300	360	660	1500	2700	4200
	Sichuan	180	300	360	720	1800	3000	4500
	Hainan	60	300	360	720	1500	2700	4200
	Anhui	180	300	360	660	1200	2700	3900
	Fujian	180	300	360	720	1500	2640	3900
	Tibet	60	300	360	660	1200	2400	3600
	Zhejiang	180	300	360	660	1500	3000	4500
	Guizhou	180	300	360	660	1200	2400	3600
	Hunan	120	300	360	720	1920	3120	4800
	Jiangsu	120	300	360	660	1200	2400	3600
	Jiangxi	180	300	360	660	1200	2400	3600
	Yunnan	60	300	390	780	1800	3000	4500
	Henan	180	300	420	720	1500	3000	4500
	Shanxi	180	300	480	900	1800	3000	4500
	Hebei	120	300	480	840	1800	3000	4500
High Tax Regions	Beijing	250	350	400	750	1600	2900	4400
	Inner Mongolia	300	360	420	900	1800	3000	4500
	Guangdong	180	360	420	720	1800	3000	4500
	Shandong	240	360	420	900	1800	3000	4500
	Xinjiang	180	360	420	720	1800	3000	4500
	Hubei	240	360	420	720	1800	3000	4500
	Guangxi	60	360	420	780	1800	3000	4500
	Shanghai	180	360	450	720	1500	3000	4500
	Tianjin	270	390	450	900	1800	3000	4500
	Liaoning	300	420	480	900	1800	3000	4500
	Gansu	240	420	480	720	1800	3000	4500
	Shaanxi	180	360	480	720	1800	3000	4500
	Jilin	240	420	480	900	1800	3000	4500
	Heilongjiang	240	420	480	900	1800	3000	4500

Note: data were obtained from the provinces’ vehicle and vessel tax charge documents.
